# Functional connectivity associated with the severity of the Anxiety-Tension stage of Emotional Burnout

**DOI:** 10.1192/j.eurpsy.2026.11761

**Published:** 2026-07-31

**Authors:** S. Tukaiev, D. Harmatiuk, A. Popov, J. M. A. Ferreira, S. Mushta, B. Palamar, I. Zyma, M. Makarchuk

**Affiliations:** 1 Educational Scientific Institute of High Technologies, Taras Shevchenko National University of Kyiv, Kyiv, Ukraine; 2 BrainPatch, Cambridge, United Kingdom; 3 Department of electronic engineering, Igor Sikorsky Kyiv Polytechnic Institute, Kyiv; 4 Faculty of Applied Sciences, Ukrainian Catholic University, Lviv, Ukraine; 5 Faculdade de Medicina, Universidade de Coimbra, Coimbra, Portugal; 6 Department of Social Medicine and Public Health, Bogomolets National Medical University; 7 Institute of Biology and Medicine, Taras Shevchenko National University of Kyiv, Kyiv, Ukraine

## Abstract

**Introduction:**

Burnout develops gradually and often goes unnoticed, with symptoms that may take years to manifest and lead to significant mental and behavioral changes. Despite its impact, the processes underlying burnout remain largely unknown due to a lack of specialized studies identifying specific biomarkers. Early detection of burnout, particularly at the critical onset of symptoms, is essential.

**Objectives:**

This study aimed to examine functional connectivity changes associated with the severity of the Anxiety-Tension stage of Emotional Burnout.

**Methods:**

A total of 752 participants, including students and staff from Taras Shevchenko National University of Kyiv (209 males, mean age = 19.2; 543 females, mean age = 18.28), were assessed using the 84-item Boyko’s Syndrome of Emotional Burnout Inventory. EEG data were recorded during a 3-minute resting state with eyes closed, and artifact-free segments from all frequency bands (0.2–45 Hz) were analyzed. Coherence were calculated, focusing on the 61–70 second segment of the recordings.

**Results:**

Coherence analysis complemented the spectral findings, highlighting alpha band synchronization in frontal and parietal areas, which supports visuospatial attention and memory processes. In contrast, desynchronization in high beta and gamma bands indicated disrupted connectivity, affecting higher-order cognitive functions.

**Conclusions:**

These findings suggest that the Anxiety-Tension stage primarily impacts processes related to short-term memory and focused attention, providing a potential framework for the early identification of burnout through neurophysiological markers.

**Disclosure of Interest:**

None Declared

